# Facilitators and barriers to integrated malaria prevention in Wakiso district, Uganda: A photovoice study

**DOI:** 10.1371/journal.pgph.0002469

**Published:** 2024-04-16

**Authors:** David Musoke, Grace B. Lubega, Filimin Niyongabo, Suzan Nakalawa, Shannon McMorrow, Rhoda K. Wanyenze, Moses R. Kamya

**Affiliations:** 1 Department of Disease Control and Environmental Health, School of Public Health, College of Health Sciences, Makerere University, Kampala, Uganda; 2 School of Interdisciplinary Health Programs, Western Michigan University, Kalamazoo, Michigan, United States of America; 3 Department of Medicine, School of Medicine, College of Health Sciences, Makerere University, Kampala, Uganda; Cheikh Anta Diop University: Universite Cheikh Anta Diop, SENEGAL

## Abstract

Malaria continues to cause significant morbidity and mortality globally, particularly in sub-Saharan Africa. Appropriate combinations of non-chemical and chemical methods of malaria vector control in the context of integrated vector management have been recommended by the World Health Organization. The aim of the study was to explore facilitators and barriers to using integrated malaria prevention in Wakiso district, Uganda. This qualitative study employed photovoice among 20 community members in Kasanje Town Council, Wakiso District. The photos taken by participants for 5 months using smartphones were discussed during monthly meetings with the researchers. The discussions were audio-recorded, and resulting data analysed using thematic analysis with the support of NVivo (2020) QSR International. Findings indicated that various conventional and non-conventional measures were being used for preventing malaria such as: insecticide treated nets; clearing overgrown vegetation; draining stagnant water; mosquito coils; smouldering of cow dung; spraying insecticides; plant repellents near houses; eating of prophylactic herbs; as well as closing doors and windows on houses early in the evening. Facilitators supporting the use of several malaria prevention methods holistically included: low cost and accessibility of some methods such as slashing overgrown vegetation; and support provided for certain methods such as receiving free mosquito nets from the government. Barriers to using several malaria prevention methods holistically included: inadequate knowledge of some methods such as housing improvement; allergic reactions to chemical-based methods such as insecticide treated nets; unaffordability of some methods such as insecticide sprays; and inaccessibility of certain methods such as body repellents. These barriers to integrated malaria prevention need to be addressed to achieve greater impact from the combination of methods in endemic communities.

## Introduction

Malaria continues to be a major public health challenge in many parts of the world particularly in sub-Saharan Africa. The disease disproportionately affects children under the age of 5 years and pregnant women, who are at higher risk of severe illness and death [1–4]. Approximately, 90% of cases and 92% of deaths due to malaria occur in sub-Saharan Africa [5]. The disease has significant economic impacts, including reduced productivity and increased healthcare costs, with an estimated total economic burden of US$ 12 billion annually in sub-Saharan Africa [6]. In Uganda, malaria remains one of the leading causes of morbidity and mortality, with an estimated 8 million cases and over 15,000 deaths reported annually [7]. A national survey estimated that malaria accounts for over 40% of outpatient visits and approximately 15–20% of hospital admissions in the country [8]. In addition, the disease is known to lead to the loss of several school and workdays and time caring for the sick, as well as vast economic burden to households [9]. Despite the implementation of various prevention strategies with a key focus on the use of long-lasting insecticidal nets (LLIN) and indoor residual spraying (IRS), the disease remains a persistent problem in the country [10]. Therefore, innovative approaches that could contribute to the reduction in the occurrence of malaria are needed in Uganda and other endemic countries.

To combat malaria, an integrated preventive approach has been recommended by leading global public health entities including the World Health Organization (WHO), which has been a key advocate for this strategy [11]. Integrated malaria prevention refers to the use of multiple, complementary strategies at households and in the community to prevent the disease. This approach recognizes that no single intervention is enough to effectively prevent malaria and aims to maximize the synergistic effect of combined methods. By combining multiple strategies, integrated malaria prevention aims to reduce transmission and ultimately reduce the burden of the disease [12–15]. The approach is effective in reducing malaria incidence and mortality, particularly when implemented in a targeted and sustained manner [16–18]. In recent years, the importance of integrated malaria prevention has been reinforced by evidence demonstrating the effectiveness of this approach in reducing the occurrence of the disease. As a result, integrated malaria prevention has become a cornerstone of global efforts to control and eliminate the disease. Indeed, many countries, including Uganda, are implementing integrated malaria prevention programmes such as combining chemical based methods such as LLINs with reducing mosquito breeding sites [19,20]. However, evidence on the use of multiple malaria prevention methods holistically and their contribution to reducing the burden of the disease is limited.

There are significant barriers to the implementation of integrated malaria prevention programmes such as limited resources and inadequate community engagement. In addition, the burden of implementing several methods and the health concerns related to chemical-based methods such as IRS and body repellents have been found to affect the integrated approach to malaria prevention [21]. Furthermore, a systematic review of global malaria prevention strategies highlighted the need to understand contextual factors such as the role of cultural and social norms, access to health services, and health literacy when implementing integrated malaria approaches in rural communities [22,23]. Consideration of these factors can increase community ownership, promote the uptake and use of multiple malaria prevention measures, and help to address barriers that may exist. This, in turn, can help to enhance the facilitators for integrated malaria preventive measures in communities [23,24]. For example, increased awareness on the various potential methods that could be used by individuals and households could enhance utilisation of the integrated approach to prevent malaria. This therefore necessitates more literature on the use of integrated malaria prevention to contribute to efforts of controlling the disease.

Most of the existing evidence on integrated malaria prevention used traditional research methodologies such as questionnaire surveys and interviews [25,26]. In these methodologies, there is little power with the participants as the researchers lead the entire processes [27]. A community-based participatory research method such as photovoice in which participants have more power in the conduct of studies was therefore needed. Photovoice puts cameras in the hands of participants which gives them the opportunity to take an active role and have influence on the study through the photographs they take [28,29]. In addition, photovoice has the potential to unveil aspects of a community that may be overlooked in traditional research methods. By allowing participants to visually express what matters most to them, researchers gain access to rich and contextually relevant data including for diseases such as malaria that might not be fully captured through other methods such as surveys or interviews [30]. Use of photovoice in malaria research would also add to evidence on use of participatory approaches in communities to reduce the burden of the disease. We therefore used photovoice to explore facilitators and barriers to using integrated malaria prevention in Wakiso district, Uganda.

## Methodology

### Study design

The study employed photovoice to explore issues related to implementation of the various methods in integrated malaria prevention in a rural, malaria endemic community in Wakiso district, Uganda. This community-based participatory research approach was carried out by 20 community members over a period of six months between 23^rd^ May and 01^st^ December 2022. The 20 participants were sufficient for the study based on existing photovoice literature [31]. The photos taken were discussed by the participants together with the researchers to explore facilitators and barriers regarding use of the integrated approach to prevent malaria.

### Study area and participants

The study was carried out in Kasanje town council, Wakiso district, a largely rural area in the central region of Uganda. The town council has one government health facility (Kasanje Health Centre III) and several private facilities including clinics, with the communities having limited access to malaria prevention and control services. The population is engaged in various economic and social activities such as brick making, crop farming, animal husbandry, petty trading, stone quarrying, and sand mining. Brickmaking and sand mining generate large pools of stagnating water and facilitate mosquito breeding in the area, hence the occurrence of malaria. Kasanje town council has a population of 29,008, with 14,597 males and 14,411 females [32]. Rural villages in the town council were purposively selected for inclusion in the study due to their higher prevalence of malaria than urban settings. These villages (cells) were from 6 wards of Jjungo, Kasanje, Makko, Bulumbu, Zziba, and Ssazi.

The 20 participants who were involved in the study were aged 18 years and above. They were selected purposively with support of local leaders in the study area based on the villages they served. The criteria for selecting the participants was provided to the local leaders by the researchers. This criteria included identifying individuals situated in various localities in the area such as those in hilly and valley settings. Other criteria used in the selection to ensure diversity included: wealth of households, gender, and occupation. Once the local leaders had identified potential study participants, the researchers reviewed and confirmed that they were suitable for the study. A diverse group of participants was selected to ensure a wide variety of perspectives and experiences on using integrated malaria prevention by different community members as was the case in our earlier photovoice studies [33–35]. Only 1 participant per village was selected for their involvement in the study.

### Training workshop and photography assignment

After recruiting the participants, a training workshop was conducted to provide the required knowledge and skills for the research such as the use and care of smartphones, as well as ethics in photography. The training also discussed various malaria prevention methods in integrated malaria prevention, and how to approach people and get consent before taking their pictures. Participants were asked to use the smart phones provided to them by the researchers to capture aspects and situations related to integrated malaria prevention in the community, including within their households. These photographs were used to facilitate the research by identifying facilitators of and barriers to using the different malaria prevention methods in their own local setting both individually and holistically. The participants were given 5 months for taking the photographs which was found to be an adequate duration for such photovoice studies [33,34]. Indeed, the duration of the study was sufficient to reach saturation as no photos on new themes emerged in the final 2 months of photography. A follow-up visit 2 weeks after commencement of photography was carried out by the researchers to ensure the assignment was being undertaken as planned. During this visit, the challenges faced by the participants with using the smart phones, among others, were addressed. Monthly meetings were held between participants and researchers to discuss the photos taken during the previous month. During the entire 5 months of photography, regular supervision of the participants by the researchers was also carried out through site visits to ensure fidelity.

### Discussing photos and data analysis

After the photographs were taken, each participant was asked to talk about all the photographs they felt were relevant to the study aim of malaria prevention in the community. Emphasis was given to what facilitated use of any of the methods, and challenges faced while attempting to use other practices at their households and in the community. Participatory analysis involved participants themselves discussing and analysing the photos taken during the monthly meetings. The discussions were conducted in *Luganda*, the local language most used in the study area. The discussion of photos over the 5-month period provided information related to facilitators and barriers to use of the various malaria prevention methods. The discussions, which were facilitated by the researchers, were audio recorded and later transcribed verbatim in the local language by one of the researchers (FN) who had experience in qualitative methods. Once the transcripts were verified, they were translated into English for analysis.

Analysis was done by the researchers (GBL, FN and DM) with vast experience in qualitative research. Thematic analysis was done with the support of NVivo (2020) QSR International. The analysis initially involved the researchers reading the transcripts several times to familiarize themselves with the data. Thereafter, words and related phrases from the transcripts were grouped to form codes, with the initial coding done by GBL and FN. Any disagreements in the generated codes were resolved by another researcher (DM). This involved a meeting where the 3 investigators met and discussed contentious codes in detail, moderated by the third researcher. Related codes were then grouped to form sub-themes, and related sub-themes grouped together to form themes. During consolidation of themes, several sub-themes were fit within the respective main themes that emerged from the data. The themes generated from the analysis were used in writing the manuscript. During manuscript writing, selected photos and quotes were used as part of the findings.

### Dissemination

On completion of the 5 rounds of photography, monthly discussions of photos and participatory analysis between participants and researchers, a dissemination workshop was held for various local stakeholders. These stakeholders included community members, local leaders, community health workers (CHWs), health practitioners, and researchers. During the workshop, a selection of the most relevant photos was showcased by the participants to share findings, experiences, and recommendations for integrated malaria prevention to the wider community.

### Ethical considerations

Ethical approval to conduct the study was obtained from Makerere University School of Public Health Research and Ethics Committee (SPH-2022-250). The research also received approval from the Uganda National Council for Science and Technology (HS2270ES). Participation in the research was voluntary, and participants provided written informed consent after an explanation of the proposed research including the anticipated risks and potential benefits before taking part. Participants were informed about ethics and safety in research including concerns in the use of photography such as getting people’s consent before taking their photos. It was also stressed during the training of participants that acceptance of a community member to take their photo would be voluntary, and refusal would not affect them in any way. A notebook was provided to each participant to record any scenario that would not be captured as a photo. For example, when someone refused to consent for a photo to be taken or when a participant did not have their smart phone at the time. Use of such a notebook ensured participants did not feel obliged to take photos of every scenario as was the case in our previous photovoice studies [36,37]. No photograph identifying individuals was used for any form of dissemination including in the publication without the consent of both the photographer and identified people. All data emanating from the study was confidentially stored by the researchers, with restricted access given to other members of the research team whenever the need arose.

## Results

The study involved 12 female participants, and the majority were farmers (14) and married (13) (Table 1). Over the 5 months, 912 photos were taken by the participants. From the ensuing discussion and data analysis emerged 3 major themes: malaria prevention methods used; facilitators of integrated malaria prevention (low cost and accessibility of methods; and support provided for certain methods); and barriers to integrated malaria prevention (inadequate knowledge and inaccessibility of some methods; allergic reactions to chemical-based methods; and unaffordability of some methods).

**Table 1:** Socio-demographic characteristics of participants.

Number	Sex	Age (years)	Occupation	Marital status
1	M	64	Farmer	Married
2	F	49	Farmer	Married
3	F	36	Farmer	Married
4	M	44	Farmer	Married
5	F	47	Farmer	Married
6	M	45	Businessman	Married
7	F	50	Farmer	Married
8	F	31	Businessman	Married
9	F	39	Farmer	Married
10	F	31	Businesswoman	Married
11	F	44	Farmer	Single
12	M	65	Businessman	Married
13	F	43	Businessman	Married
14	M	32	Businessman	Single
15	M	32	Farmer	Married
16	M	54	Farmer	Married
17	F	47	Farmer	Married
18	M	66	Farmer	Married
19	F	39	Farmer	Married
20	F	57	Farmer	Single

### Malaria prevention methods used

Findings revealed that various conventional and non-conventional measures were used for preventing malaria in the study area. Among the conventional methods, sleeping under LLINs was the most prominent. Other well-known methods being used included: slashing of overgrown vegetation surrounding houses; draining of stagnant water; screening of windows; closure of windows and doors before 6:00 pm; use of mosquito coils; spraying of insecticides; and IRS. Participants also mentioned that prophylaxis with antimalarials was being used among pregnant women to prevent malaria. According to the participants, this medication was predominantly given to pregnant women as part of their antenatal care visits to health facilities. Mosquito coils were particularly favoured by many households that were not using other methods.

“*A mosquito coil is one of those materials that release a scent that repels mosquitoes*. *It is lit and the smoke repels mosquitoes*. *It is always appropriate to light it before people plan to go to sleep*. *It helps so that by the time people go to sleep*, *the house is free from mosquitoes*.*”* Male, Participant 14, Meeting 2

Regarding the non-conventional methods, participants mentioned the use of plant repellents around houses and eating of herbs they said were prophylactic. Examples of common plant repellents included neem tree, rosemary, and lemon eucalyptus. Participants stated that they grew these plants near their homes which repelled mosquitoes through their scent, while others mentioned that they burnt plant repellents inside their houses before going to bed. According to the participants, the smoke from the burning repelled mosquitoes. In addition, smouldering of cow dung inside and outside houses was used to prevent mosquitoes from biting household members. Another non-conventional method was the eating of herbs that were believed to have malaria-inhibiting properties. Examples of such herbs included: *Vernonia amygydalina*, *Aristolochia littoralis*, *Gynandropsis gynandra*, and *Cleme gynandra*. The herbs were cooked and eaten as vegetables during meals or added to sauces.

“*Whenever my parent cooks vegetables*, *she adds leaves of a local medicinal herb*. *When she also cooks groundnut paste*, *she adds in another herb*. *They* [herbs] *are very bitter and in most cases*, *we never wanted to eat the food but were forced to*. *She used to explain to us that she was protecting us from malaria and indeed we did not fall sick from the disease*.” Male, Participant 16, Meeting 4

### Facilitators of integrated malaria prevention

Facilitators to using several malaria prevention methods holistically were captured through photos and accompanying narratives. These included low cost and accessibility of methods, and support provided for certain methods.

### Low cost and accessibility of methods

**Fig 1:**
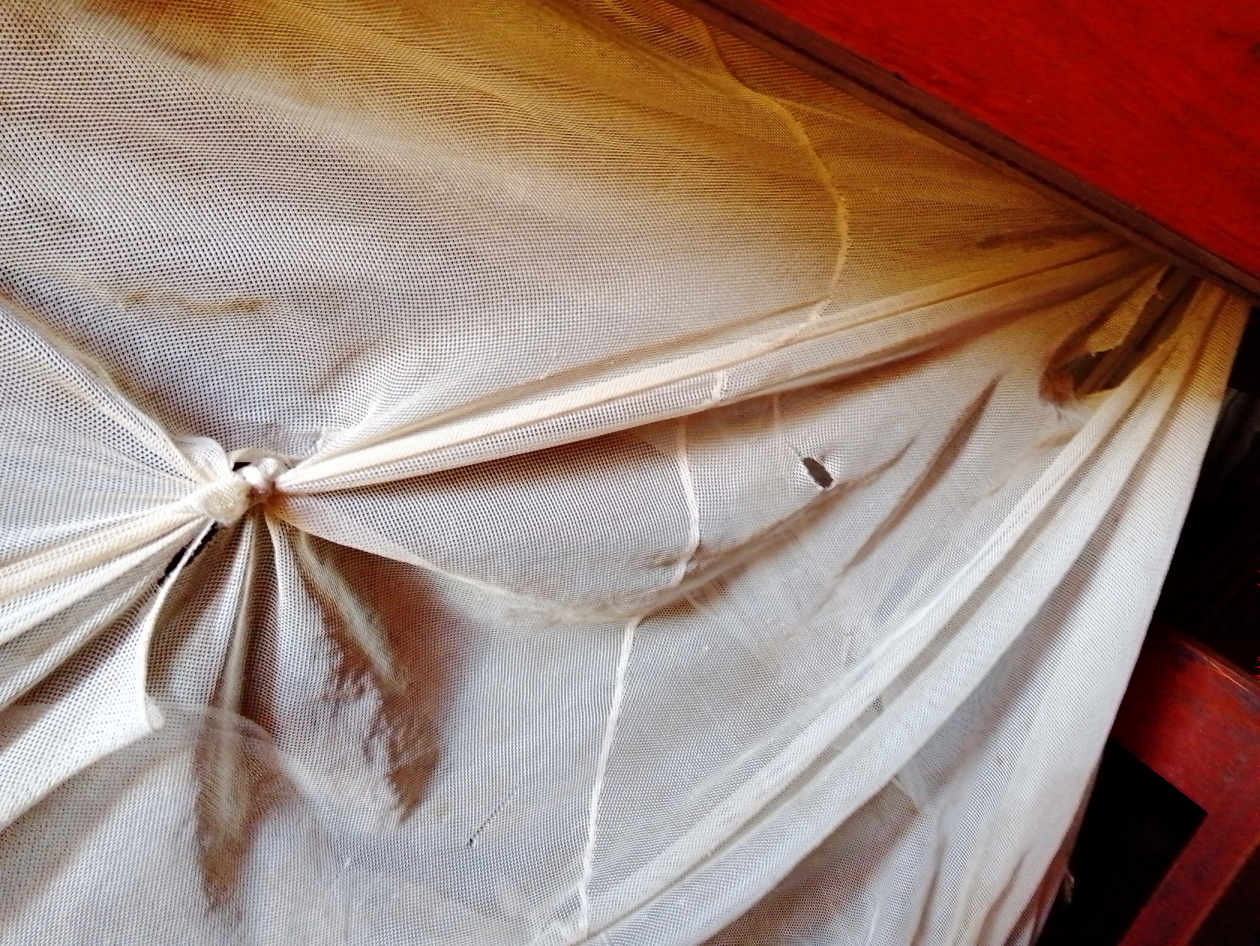
A torn LLIN with a tied knot to prevent entry of mosquitoes.

According to the participants, closure of windows and doors before 6:00pm, and slashing overgrown vegetation could easily be used with other methods. This was because both methods could be done daily without any costs involved. Participants also agreed that the repairing of LLINs that had holes could be done easily once community members had a needle and thread. Having a sewing kit was therefore considered a necessity for many homes. Other participants mentioned that if their mosquito nets got torn, they would tie the part with the hole at no cost (Fig 1).

**Fig 2:**
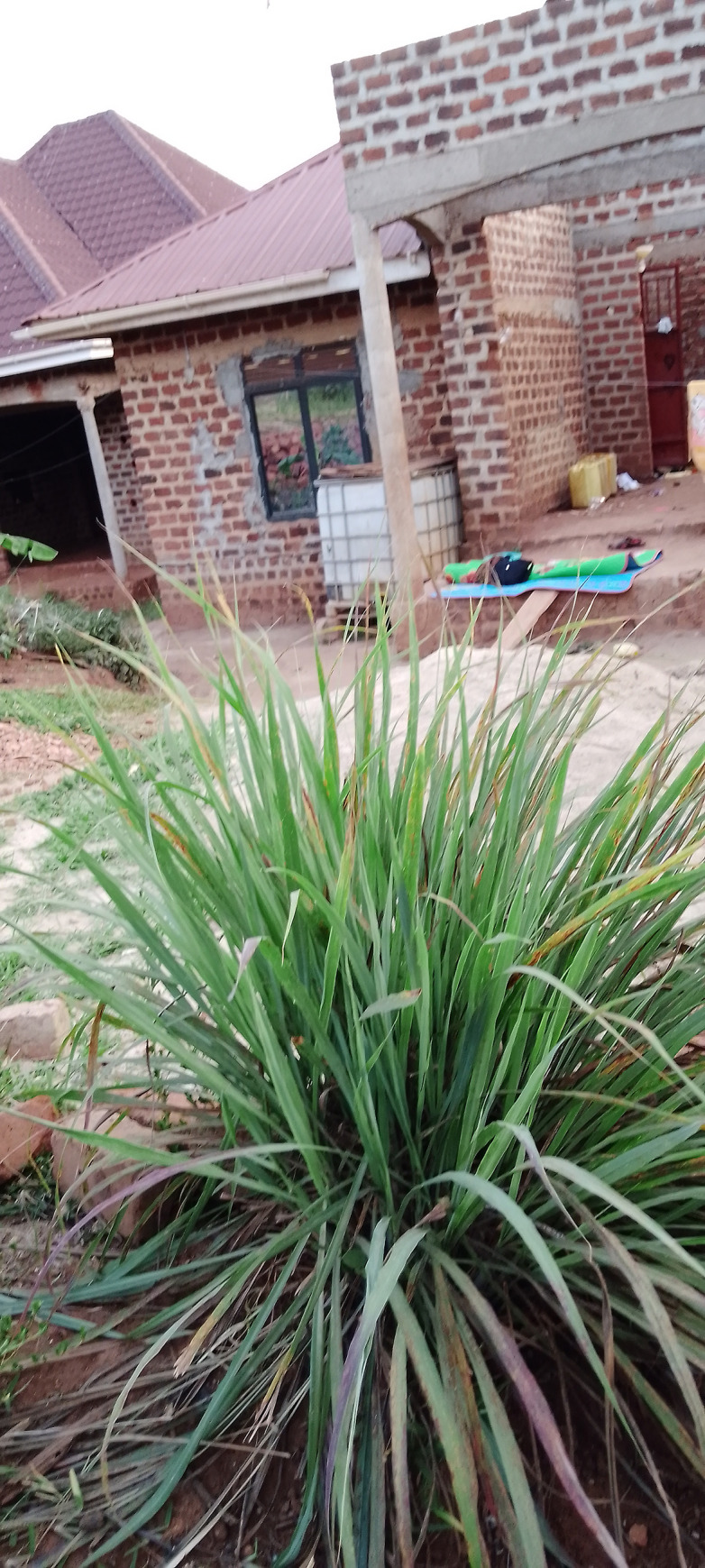
A local plant used as a mosquito repellent near a community member’s home.

The use of plant repellents was also found to be cheap because the repellent seeds were usually bought at once and planted around compounds (Fig 2). Some participants mentioned that at times these plants grew on their own, while others were found in neighbouring shrubs hence participants could easily use them at no cost. Other participants highlighted that the plant repellents were also versatile and multi-purposeful. For example, having plant repellents around homes beautified the compound and conserved the environment.

Participants also mentioned that some methods such as the use of mosquito coils were readily available in local shops at a low cost. Among participants who reared animals, the smouldering of cow dung was one method that they easily used as the material was available most of the time. Given that most participants preferred to keep animals near their houses which they mentioned attracted mosquitoes, implementing the strategy of burning dry cow dung in addition to using LLINs was considered a viable option for holistic malaria prevention.

### Support provided for certain methods

According to the participants, several methods could be used holistically if community members were regularly educated and reminded of how to integrate different malaria prevention methods. One participant reported that after showing their community members the importance of slashing the compound regularly in addition to using LLINs, they started practicing both methods. Participants also mentioned that the Government of Uganda through the Ministry of Health provided free LLINs regularly which could be used together with other methods such as spraying insecticides, clearing overgrown vegetation, and draining stagnant water.

“*There was a lady in that home*. *The interesting thing is that where you see the little green now was a bush*. *But of recent*, *the lady had cleared the bush after our conversation*. *She had a good compound but due to the long grass previously*, *mosquitoes were very many at her home at night*. *She also informed me that she didn’t know mosquitoes were coming from her compound because it was bushy*. *She had thought they come from some other places*.*”* Male, Participant 15, Meeting 4

### Barriers to integrated malaria prevention

Barriers to using several malaria prevention methods holistically included: inadequate knowledge and inaccessibility of some methods; allergic reactions to chemical-based methods; and affordability of certain methods.

### Inadequate knowledge and inaccessibility of some methods

Participants highlighted that some community members were not aware of certain malaria prevention methods such as the use of body repellents. In addition, participants mentioned that they did not know about some malaria prevention methods that were presented by their colleagues during the monthly meetings such as electric mosquito traps. Other participants stated that many community members did not know the importance of using multiple methods. For example, many participants reported that some of the people they interacted with during the study did not know that housing improvements such as properly fitting windows and doors, filling of holes in walls, placement of glass panes in windows and doors, and the use of screens in windows reduced entry of mosquitoes in houses. Participants also revealed that body repellents and electric traps were not readily accessible within their communities thus prevented them from using these methods alongside other measures. For example, many participants stated that they had not seen body repellents in their communities, despite being aware of the possible use of vaseline-based repellents especially while outdoors at night.

“*When I was moving around*, *I realised that people have no knowledge and need to be taught about other methods apart from the mosquito net usage and closing doors early*. *Therefore*, *people need to be taught to understand the different ways that can be used to prevent malaria especially housing improvement*.*”* Male, Participant 5, Meeting 3

### Allergic reactions to chemical-based methods

Participants mentioned that some of the malaria prevention methods such as the use of LLINs, IRS, and insecticide sprays caused allergic reactions such as skin irritation and discomfort among users. Therefore, some people could not integrate them with other prevention methods. Other participants stated that smouldering of plant repellents and cow dung sometimes caused discomforts such as respiratory irritation among some people, thereby preventing their holistic use. According to the participants, it was very common for pregnant women to have shortness of breath when inside houses that had been sprayed with insecticides.

“There was a lady who was seated in the bedroom at night without the mosquito net fitted on the bed. When I asked her why she was not using the net, she told me that it disturbs her and gets breathless when she sleeps under it.” Female, Participant 19, Meeting 5

### Unaffordability of some methods

**Fig 3:**
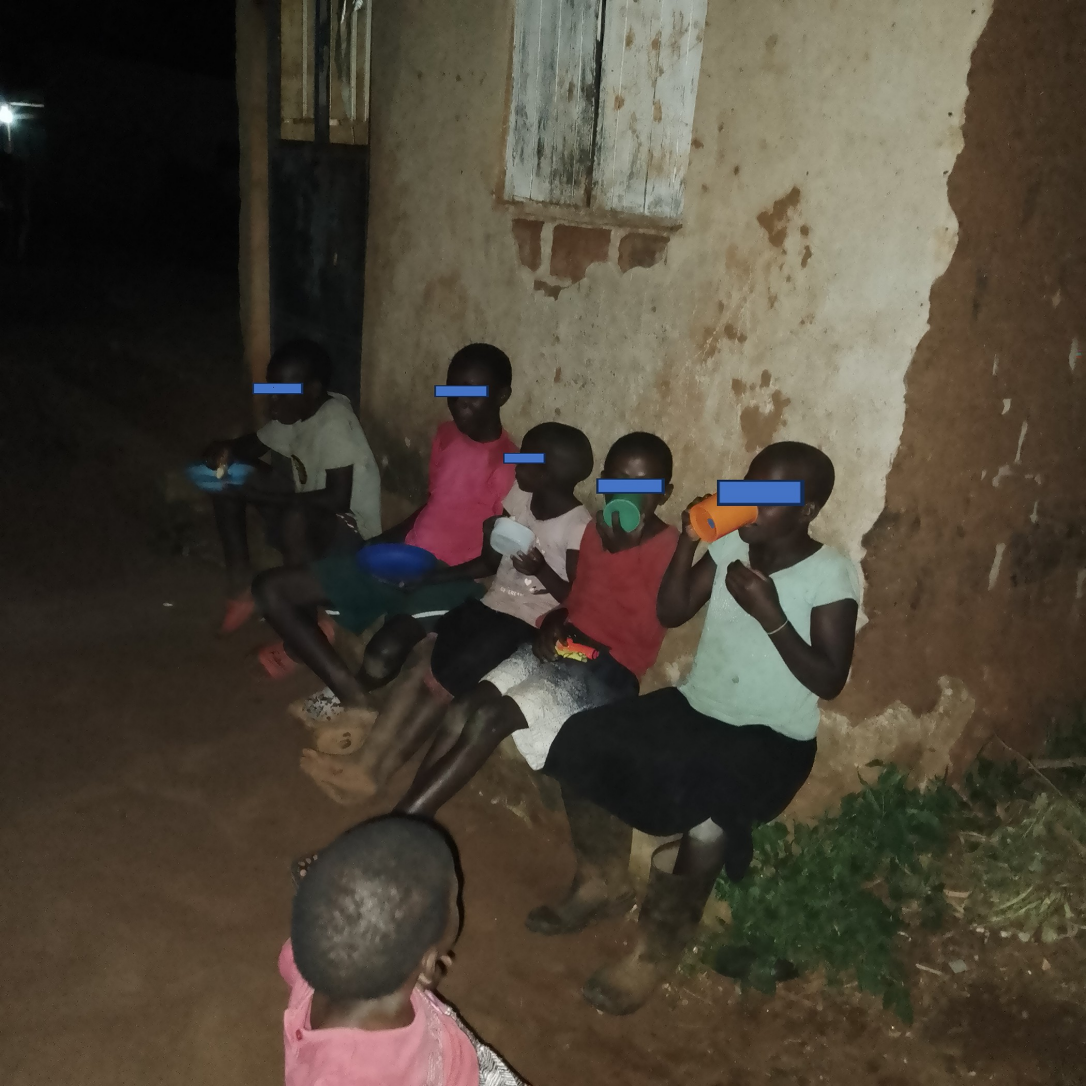
Children eating dinner outside the house (due to lack of light indoors) which exposed them to mosquitoes.

Participants stated that some malaria prevention methods were expensive, especially those that necessitated regular buying. Some participants said that members of their community did not have the money to buy electric mosquito traps and body repellents. Furthermore, the use of electric mosquito traps was linked to increased electricity costs due to routine charging. It was therefore very common for people including children to eat dinner and do homework outside the houses at night due to adequate lighting indoors which exposed them to mosquito bites. This was because household heads could not always afford to purchase kerosene for lamps and therefore used natural light from the moon (Fig 3). Indeed, all the participants agreed that poverty was a major barrier to the integration of the different malaria prevention methods in the community.

**Fig 4:**
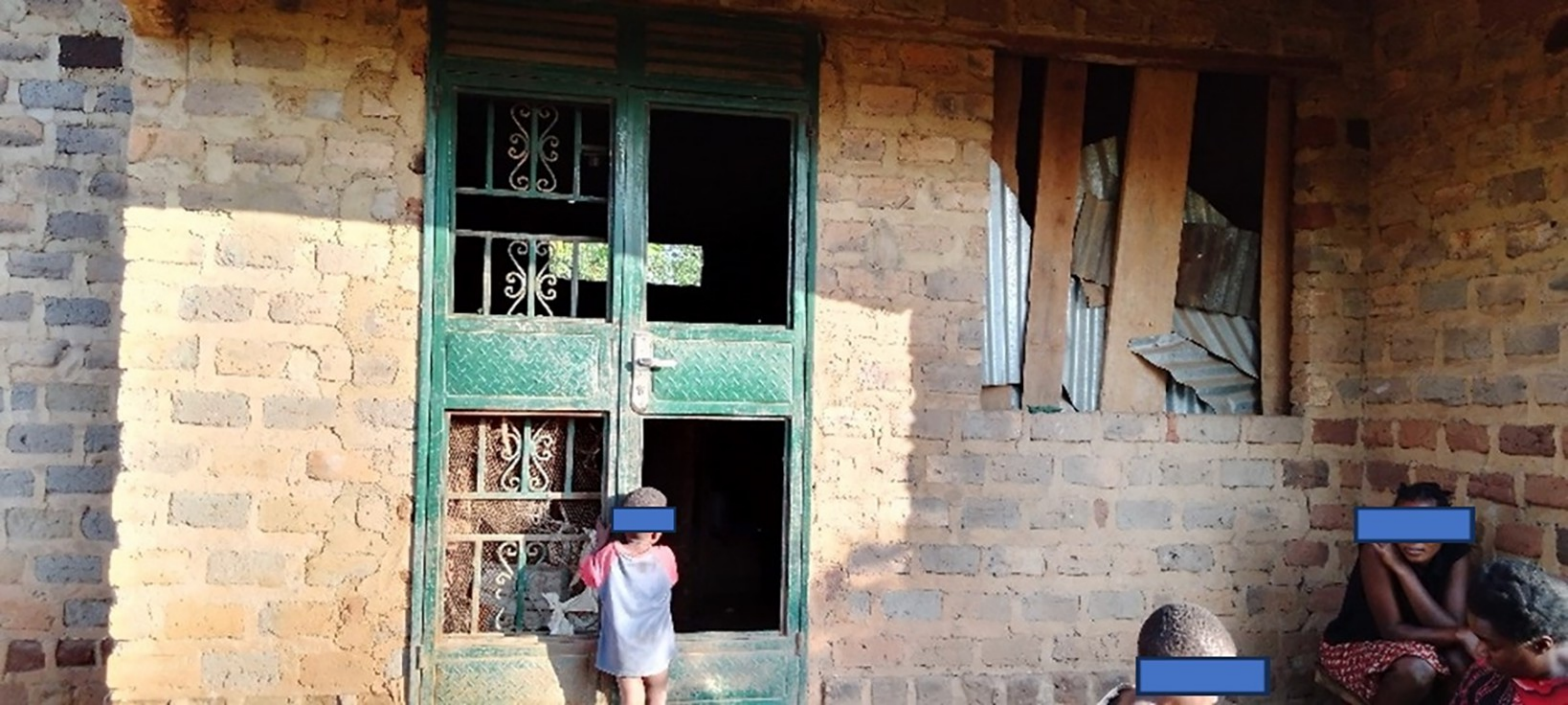
A house with missing window and door panes. Iron sheets and pieces of wood had been placed in the window which left gaps that permitted mosquito entry.

According to the participants, money was a necessity for implementing different methods such as supporting housing improvement with the right quality of building materials and replacing broken glass window and door panes. Participants established that many people were sleeping in incomplete and poorly built houses because they lacked funds. Indeed, many participants captured photos of houses with poorly fitted doors and windows, those with missing window panes, those with holes in their walls, those with screening in vents, and others with windows and doors having spaces in them that allowed mosquito entry. The use of cloth materials, iron sheets, and rods to cover the window and door spaces as a way of improvising for the lack of standard panes on houses was common (Fig 4).

## Discussion

This qualitative photovoice study presented community members with the opportunity to assess facilitators and barriers to holistic malaria prevention. It also explored realistic and feasible ways for integration of malaria prevention practices, both conventional and non-conventional, thereby contributing to reducing the burden of the disease. The prominence of non-conventional methods in the study such as plant repellents, smouldering animal dung, and prophylactic herbs are important findings that should be explored when discussing holistic malaria prevention in rural settings. Indeed, these methods may require further investigation to better appreciate their contribution to malaria control in Uganda and elsewhere. If these methods are proven to be effective, they could complement existing malaria control strategies locally and globally. Facilitators of integrated malaria prevention such as those related to low cost and availability of various methods could be harnessed to enhance integrated control of mosquitoes in communities. Strategies to address the barriers to using multiple methods such as inadequate knowledge and inaccessibility of some methods need to be addressed as part of national malaria control efforts. These findings provide evidence on integrated malaria prevention interventions in the context of a rural setting in Uganda which could be used to support future research as well as policy and practice related to integrated vector management as recommended by the WHO [37].

Many non-conventional methods established in our study such as smouldering animal dung and use of prophylactic herbs were considered cheap and easily accessible by the community which facilitated integration. Rural communities are characterised by vast vegetation cover which facilitates growth of plant repellents and rearing of animals which provide cow dung. Herbs that were used for malaria prophylaxis can easily be collected from gardens, bushes, as well as nearby shrubs and neighbourhoods [38]. Previous qualitative and quantitative studies in Uganda [38,39], Kenya [40–42], West Africa [43], Tanzania [44], and India [45] have all reported burning of cow dung and plant repellents to prevent mosquito bites and entry into houses. Eating herbal plants in groundnut paste to prevent malaria has also been documented elsewhere [38]. Research on the effectiveness, efficacy, and safety of non-conventional methods such as prophylaxis with herbs and burning of cow dung, alongside conventional ones should be conducted to potentially contribute to national and global malaria control efforts.

Support from the government and health workers was seen as another facilitator to integrated malaria prevention in our study. Other quantitative and qualitative studies we have recently conducted documented the importance of government support in malaria prevention especially in rural settings [46,47]. Future provision of free LLINs to the population by MOH is likely to further facilitate use of multiple malaria prevention methods at households. Integrated malaria prevention requires regular education and reminding community members of how to integrate the various methods [48]. An earlier pilot project on malaria prevention showed that households observed various practices with support from CHWs who carried out regular community sensitization on the different methods [49]. CHWs, who spend vast time with members of the community, could spearhead campaigns of carrying out routine health education on using multiple methods to prevent malaria. Indeed, CHWs are known to play an instrumental role in health promotion particularly in rural settings.

In our study, many of the participants and community members did not know the importance of using several malaria prevention methods. This finding is similar to previous quantitative studies where few methods were being integrated due to lack of knowledge [45–47,50]. Similar to our study, knowledge and use of LLINs was prominent in previous qualitative and quantitative studies including in Kenya and Tanzania [40,44,47,49]. Adequate knowledge on LLINs among populations may be attributed to the mass campaigns on use of mosquito nets including by the Ugandan Ministry of Health [8]. Future malaria initiatives should also emphasize the importance of using several other methods alongside LLINs to increase knowledge of other malaria prevention practices which could contribute to increased utilisation. Furthermore, holistic malaria prevention activities can also support the shortfalls in use of LLINs such as low uptake and sustainability in communities [20].

Participants mentioned that some malaria prevention methods such as body repellents were inaccessible in their communities. A quantitative study done in Ethiopia found that 29.9% of the participants did not use malaria prevention methods due to accessibility challenges [50]. Inaccessibility of malaria services undermines collective efforts to eradicate the disease by 2050 [51]. Furthermore, some of the malaria prevention methods used such as LLINs (in cases where free ones are not provided by the government), electric mosquito traps, and body repellents were considered as expensive. Views of some malaria prevention methods being unaffordable have been expressed in other qualitative and quantitative studies [41,45,47,50]. This could be because rural communities have many competing financial priorities yet they are generally poor [41]. The use of electric mosquito traps is best suited for communities with an electric power grid, which may not be the case with many households in rural areas. Furthermore, the few houses that are connected to the electricity in rural settings cannot afford the high-power tariffs hence rendering electric devices unsustainable. Given that the average number of persons in households in Uganda is 5 [52], frequent purchase of body repellents in rural areas is unsustainable. Addressing the social determinants of health in rural communities such as poverty is likely to improve malaria prevention practices in the short and long term.

Poor housing quality was another barrier to integrated malaria prevention. Concerns of mosquito entry in houses due to open eaves, as well as gaps in doors and windows have also been documented in several countries including Gambia [53] and Ethiopia [54]. In our study, participants stated that people were sleeping in incomplete and poorly built houses because of the costs involved. It is characteristic of rural houses to have open eaves and incomplete walls which allows entry of mosquitoes [41]. An earlier study on reducing malaria by mosquito proofing suggests that the importance of screening houses has been ignored in many areas [55]. In our study, household members improvised with the use of iron sheets and cloth materials to provide temporary screening in windows and doors. However, these materials are not effective in preventing mosquito entry into houses. Indeed, recent case studies in Uganda and Tanzania have recommended improving the design and structures of houses to reduce mosquito entry [48].

Despite participants agreeing that the use of LLINs, IRS, and spraying of insecticides were core methods to integrated malaria prevention, concerns were expressed of these chemical-based methods being linked to allergic reactions and other negative effects on health. Quantitative studies elsewhere have documented allergic reactions such as skin irritation and discomfort due to the use of these methods [56,57] even among pregnant women [58]. However, it should be noted that such reactions are usually short-term [59,60] and are mostly caused by failure to observe recommended guidelines of hanging nets outside for 24 hours before usage [61]. In addition, staying outside the house for at least 2 hours after IRS is complete to allow the insecticide to dry could minimise side effects of the intervention [62]. Continuous health education on the recommended usage of chemical-based malaria prevention methods in homes by health workers and CHWs is necessary especially during community LLIN and IRS campaigns.

The use of photovoice in our study not only allowed participants to identify facilitators and barriers to holistic malaria integration but also enabled them to take leadership in identifying malaria prevention practices within their communities. Engaging 20 participants who were diverse enabled the collection of a wide range of perspectives and experiences. However, our study was carried out in only one predominantly rural area in Wakiso district hence the findings may be different within other geographical contexts hence not generalisable to other settings. In addition, evaluation of the community’s knowledge and practices on malaria prevention following the participatory approach was not assessed. Future studies on integrated malaria prevention including non-conventional methods using both qualitative and quantitative methods are recommended in peri-urban and urban settings in Uganda.

## Conclusion

Several facilitators to using integrated malaria prevention were found such as low cost and accessibility of several methods, and support provided for certain methods. However, inadequate knowledge, inaccessibility, and unaffordability of some methods, as well as allergic reactions to chemical methods impeded the use of several malaria prevention methods holistically. The findings from the study could support future research, as well as inform policy and practice related to integrated vector management.

## Supporting information

S1 Data Study dataset. (PDF)
