# Supplementary material for: Facilitators and barriers to integrated malaria prevention in Wakiso district, Uganda: A photovoice study
Source: PLOS Glob Public Health. 2024 Apr 16;4(4):e0002469. doi: 10.1371/journal.pgph.0002469 (PMC11020531; doi:10.1371/journal.pgph.0002469)
Supplement: S1 Data — (PDF) [file pgph.0002469.s001.pdf]

EDCTP malaria project

Codes

Name	Description	Files	References
**Barriers to integration of malaria preventive methods**		**5**	**205**
Allergic reactions and discomfort to chemical methods	Shortness of breath, skin irritation, due to insecticide, repellents, mosquito nets and smoking; being burnt/ fires due to mosquito nets	5	25
Food security vs malaria prevention	Planting of food crops near the house due to limited space, keeping of animals near the house to avoid theft, yet some crops (maize) and animals attract mosquitoes	3	9
Inaccessibility of some methods	Not readily available e.g. electric mosquito traps and mosquito coils, Vaseline /jelly repellents,	3	7
Inadequate knowledge on some methods	On use and application of different methods; on types of methods e.g. proper housing construction, use of repellents, screening of houses	5	24
ITN to people ratio	Few nets in homes, prioritization of nets based on vulnerability	5	8
ITNs used elsewhere	In flower beds and plantations	2	4
Landlord and land ownership versus environmental protection	No control over activities on rented land or / adjust neighbouring land. Limitations on house rights such as replacement of broken windows or glass	2	5
Inevitable exposure in early or late morning hours due to livelihood	Late evening and night jobs (night watchmen, defence officers, food handlers, health workers, shop attendants, fishermen, brick layers). School night activities (homework and preps) Early morning jobs (digging, animal feeding, shop attendants), Leisure activities at night (watching TV, listening	5	51

Name	Description	Files	References
(mosquitoes bite between dusk 5:00 pm to 7:00 pm and dawn 5:00 am to 6:00 am)	to radios, and clubbing), social activities overnight prayers, burials and vigils		
Negligence	People are lazy, don't create time for mosquito prevention, women (pregnant) lack support from husbands	5	19
Politics	Beliefs that preventive methods have a political incentive	2	7
The unaffordability of some methods and poverty	Failure to purchase ITNs, complete dose malaria medication, repellents, screens, replacement of windows and door glasses, no transport to HC	4	37
Use of one method only	The belief that one method is enough to control malaria	5	9
**Conventional malaria prevention methods**		**4**	**116**
Conventional treatment of malaria	Use of antimalarials, test and treat, prophylaxis for pregnant mothers	4	8
Health education by VHTs as a result of the photovoice study	Health education on the Use of ITNs, Environment -pouring of stagnant, cleaning of compounds, slashing bushy areas, blocking of water tanks, septic tanks, bathroom soak pits. Treatment - full dose completion, visiting of the HC. Close of windows, wearing of long sleeved shirts and trousers, use of repellents, use of insecticides	4	64
Use of ITNs/sleep under a mosquito net	Use of mosquito nets	4	14
Proper housing construction and house screening	Screens on windows, glass on windows, doors and louvres	3	4
Sanitation and proper housing keeping Environment clean up [Covering stagnant water and tanks]	Good environmental hygiene, cleaning of drainage channels, cutting of bushes, long grass, closure of windows and doors at 6:00pm, and slashing of compounds/bushes	4	12

Name	Description	Files	References
**Examples of integration of malaria preventive methods**	**Existing within the communities and possible ones constructed by VHTs after participating in the photovoice study (based on outdoor and indoor presence of people and activities)**	**4**	**69**
**Facilitators to the integration of malaria preventive methods**		**5**	**25**
Convenience and safety	Believed to be safe and convenient	3	5
Durability	Method can be used for more years	2	5
Government support	Free nets	2	4
Low cost	Repair of ITNs, availability of local herbs	2	11
**Non-conventional malaria prevention methods**		**5**	**95**
Indoor residual spraying of Insecticide and pesticides	Rocket, pepper, chemicals for indoor spraying,	3	12
Wearing of long-sleeved shirts and trousers	Clothing covering body parts	2	2
Natural and manufactured mosquito repellent [Smouldering of cow dung and repellents / planting of repellent plants]	Jelly repellents smeared on the body, planted/fenced plant repellents around the house which repel mosquitoes through their scent. Burning/smoking of awudi, mosquito coils, rubbish, cow dung, lemon eucalyptus, coffee peelings, sticks, furniture wastes, and rosemary which repel mosquitoes through smoke and smell	5	42
Tradition prophylaxis [Drinking or eating of preventive local remedies/ herbs]	Drinking or eating herbs for prophylaxis	3	14

Name	Description	Files	References
Malaria treatment with local herbs	Used as first aid before going to HCs, used as an alternative to conventional medicine because its cheap and readily available, can cure other diseases in addition to malaria. Concerns of prolonged use and resistance	4	38
Mixing local medicine with conventional	Mixing local and local or local and conventional medicine	3	13
**Bad practices that can lead to malaria**		**5**	**107**
Bushy environment and poor sanitation	Houses surrounded by bushes, weeds, grass, maize plantation, dirty compounds, Herbal medicine that is bushy	5	30
ITNs not being used		4	5
Poor housing and house screening	No glasses in windows and doors, windows and doors hanging, unfinished houses with holes, screens made of cloth instead of wire mesh which leads to poor ventilation	5	37
Permanent stagnant water	Swampy areas, pots, ditches, broken containers like flower pots, water bottles, brick making bonds, fish ponds, open water tanks, grassy drainages	5	35
